# Lack of association between *PKLR *rs3020781 and *NOS1AP *rs7538490 and type 2 diabetes, overweight, obesity and related metabolic phenotypes in a Danish large-scale study: case-control studies and analyses of quantitative traits

**DOI:** 10.1186/1471-2350-9-118

**Published:** 2008-12-26

**Authors:** Camilla Helene Andreasen, Mette Sloth Mogensen, Knut Borch-Johnsen, Annelli Sandbæk, Torsten Lauritzen, Katrine Almind, Lars Hansen, Torben Jørgensen, Oluf Pedersen, Torben Hansen

**Affiliations:** 1 Steno Diabetes Center, 2820 Gentofte, Denmark; 2 Novo Nordisk A/S, Medical and Science, Development Projects, 2880 Bagsværd, Denmark; 3 Research Centre for Prevention and Health, Glostrup University Hospital, 2600 Glostrup, Denmark; 4 Faculty of Health Science, University of Aarhus, 8000 Aarhus, Denmark; 5 Department of General Practice, University of Aarhus, 8000 Aarhus, Denmark; 6 Bristol-Meyers Squibb & Co, Discovery Medicine and Clinical Pharmacology, CV Metabolic Diseases, 08543-4000 Princeton NJ, USA; 7 Faculty of Health Sciences, University of Copenhagen, 2200 Copenhagen, Denmark; 8 Faculty of Health Sciences, University of Southern Denmark, 5000 Odense, Denmark

## Abstract

**Background:**

Several studies in multiple ethnicities have reported linkage to type 2 diabetes on chromosome 1q21-25. Both *PKLR *encoding the liver pyruvate kinase and *NOS1AP *encoding the nitric oxide synthase 1 (neuronal) adaptor protein (CAPON) are positioned within this chromosomal region and are thus positional candidates for the observed linkage peak. The C-allele of *PKLR *rs3020781 and the T-allele of *NOS1AP *rs7538490 are reported to strongly associate with type 2 diabetes in various European-descent populations comprising a total of 2,198 individuals with a combined odds ratio (OR) of 1.33 [1.16–1.54] and 1.53 [1.28–1.81], respectively. Our aim was to validate these findings by investigating the impact of the two variants on type 2 diabetes and related quantitative metabolic phenotypes in a large study sample of Danes. Further, we intended to expand the analyses by examining the effect of the variants in relation to overweight and obesity.

**Methods:**

*PKLR *rs3020781 and *NOS1AP *rs7538490 were genotyped, using TaqMan allelic discrimination, in a combined study sample comprising a total of 16,801 and 16,913 individuals, respectively. The participants were ascertained from four different study groups; the population-based Inter99 cohort (*n*_*PKLR *_= 5,962, *n*_*NOS1AP *_= 6,008), a type 2 diabetic patient group (*n*_*PKLR *_= 1,873, *n*_*NOS1AP *_= 1,874) from Steno Diabetes Center, a population-based study sample (*n*_*PKLR *_= 599, *n*_*NOS1AP *_= 596) from Steno Diabetes Center and the ADDITION Denmark screening study cohort (*n*_*PKLR *_= 8,367, *n*_*NOS1AP *_= 8,435).

**Results:**

In case-control studies we evaluated the potential association between rs3020781 and rs7538490 and type 2 diabetes and obesity. No significant associations were observed for type 2 diabetes (rs3020781: *p*_AF _= 0.49, OR = 1.02 [0.96–1.10]; rs7538490: *p*_AF _= 0.84, OR = 0.99 [0.93–1.06]). Neither did we show association with overweight or obesity. Additionally, the *PKLR *and the *NOS1AP *genotypes were demonstrated not to have a major influence on diabetes-related quantitative metabolic phenotypes.

**Conclusion:**

We failed to provide evidence of an association between *PKLR *rs3020781 and *NOS1AP *rs7538490 and type 2 diabetes, overweight, obesity or related quantitative metabolic phenotypes in large-scale studies of Danes.

## Background

Type 2 diabetes (T2D) is a complex metabolic disease, where several tissues and organs, including pancreatic β-cells, skeletal muscle, adipose tissue, liver and the central nervous system have been suggested to be directly or indirectly involved in the pathogenesis [1].

Several independent studies have shown evidence for linkage between chromosome 1q21-25 and T2D in multiple ethnicities [2-14]. Both *PKLR *encoding the liver pyruvate kinase and *NOS1AP *encoding the nitric oxide synthase 1 (neuronal) adaptor protein (CAPON), are located in the 1q21-25 region and are therefore positional candidate genes for T2D susceptibility. The puryvate kinase enzyme catalyses the last step in glycolysis converting phosphoenolpyruvate to pyruvate under the generation of ATP. *PKLR *is, in addition to the liver, expressed in pancreatic β-cells, the kidneys and the small intestine [15], and its expression is upregulated by glucose through a carbohydrate response element in the promoter [16]. Moreover, a binding site for hepatocyte nuclear factor 1-α is located in the *PKLR *promoter and patients with maturity-onset diabetes of the young type 1 and 3 show decreased expression of the gene [17,18]. Hence, *PKLR *is a strong biological candidate gene for impaired blood glucose regulation and thus T2D. The CAPON protein binds nitric oxide synthase, which results in downregulation of N-methyl-D-aspartate receptor-mediated glutamate signalling [19], however, the link between dysfunctional CAPON protein and T2D is as yet unexplained.

A substantial number of genes, in this very gene-dense 1q21-25 region, have already been investigated for susceptibility to T2D, however, none have so far explained the observed linkage [1]. As a part of The International Type 2 Diabetes 1q Consortium 5,285 single-nucleotide polymorphisms (SNPs), covering 22.7 Mb of the 1q linkage region were genotyped in 1,000 cases and 1,198 matched controls from four different European-descent populations.

Two SNPs, rs3020781 in *PKLR *and rs7538490 in *NOS1AP *were reported to associate with T2D. Applying an additive model the C-allele of *PKLR *rs3020781 associated with T2D with an odds ratio (OR) of 1.33 [1.16–1.54] (*p *= 1·10^-6^), and under a dominant model the T-allele of *NOS1AP *rs7538490 associated with T2D with an OR of 1.53 [1.28–1.81] (*p *= 2·10^-6^)[38]. *PKLR *has previously been examined in two independent studies, where four SNPs, (rs3020781, rs2071053, rs1052176, rs1052177), showed association with T2D when analysing a total 909 individuals of European descent [20,21]. No further association studies regarding the role of *NOS1AP *in T2D pathogenesis have been performed.

The aim of the present study was to validate the association of *PKLR *rs3020781 and *NOS1AP *rs7538490 with T2D. In addition we intend to expand with analyses of overweight and obesity and the relationship with diabetes-related metabolic quantitative phenotypes.

## Methods

### Subjects

*PKLR *rs3020781 and *NOS1AP *rs7538490 were successfully genotyped in 16,801 and 16,913 Danes, respectively, involving four study groups 1) the population-based Inter99 cohort (ClinicalTrials.gov ID no: NCT00289237) (*n*_*PKLR *_= 5,962, *n*_*NOS1AP *_= 6,008), with an average age of 46 ± 8 years and a mean BMI of 26.2 ± 4.6 kg/m^2^, sampled at the Research Centre for Prevention and Health [22] 2) unrelated T2D patients (*n*_*PKLR *_= 1,873, *n*_*NOS1AP *_= 1,874), with an average age of 62 ± 11 years and a mean BMI of 30.0 ± 5.6 kg/m^2^, sampled through the out-patient clinic at Steno Diabetes Center 3) a population-based group of unrelated middle-aged individuals (*n*_*PKLR *_= 599, *n*_*NOS1AP *_= 596), with an average age of 59 ± 8 years and a mean BMI of 26.5 ± 4.2 kg/m^2^, examined at Steno Diabetes Center 4) the ADDITION Denmark screening study cohort (ClinicalTrials.gov ID no: NCT00237548) (*n*_*PKLR *_= 8,367, *n*_*NOS1AP *_= 8,435), with an average age of 60 ± 7 years and a mean BMI of 28.6 ± 4.9 kg/m^2^, sampled by Department of General Practice at University of Aarhus [23]. The different study groups are further described in Additional file 1. In study group 1 and 3 all non-diabetic individuals underwent a standard 75 g oral glucose tolerance test (OGTT) and only glucose-tolerant and normoglycaemic individuals were included as control subjects in the case-control study of T2D. Analyses of quantitative metabolic phenotypes were performed in the population-based Inter99 cohort exclusively, excluding T2D patients receiving pharmalogical treatment. Informed written consent was obtained from all individuals before participation. The studies were approved by the regional Ethical Committees (Ethics Committee, Copenhagen County for study group 1, 2 and 3 and Ethics Committee, Aarhus County for study group 4) and were in accordance with the principles of the *Helsinki Declaration*. T2D and normal glucose tolerance (NGT) were defined according to the World Health Organization [24].

### Biochemical and anthropometrical measurements

In all study groups body weight and height were measured in light indoor clothes and without shoes [22,23]. In study groups 1 and 3 serum insulin and plasma glucose were measured at fasting and 30 and 120 minutes after an OGTT. Serum insulin levels excluding des(31,32)- and intact proinsulin were measured using the AutoDELFIA insulin kit (Perkin-Elmer, Wallac, Turku, Finland). Plasma glucose was analysed using a glucose oxidase method (Granutest; Merck, Darmstadt, Germany) [25]. The homeostasis model assessment of insulin resistance (HOMA-IR) was calculated as fasting plasma glucose (mmol/l) multiplied by fasting serum insulin (pmol/l) divided by 22.5. The BIGTT insulin sensitivity index (BIGTT-S_i_) and BIGTT acute insulin response (BIGTT-AIR) were calculated as described [26,27].

### Genotyping

*PKLR *rs3020781 and *NOS1AP *rs7538490 were genotyped using Taqman allelic discrimination (KBioscience, Herts, UK). Discordances between 1,202 random duplicate samples were 0.1% and 0.2%, respectively, and the genotyping success rates were 96.3% and 96.8%, respectively. Both genotype groups obeyed Hardy-Weinberg equilibrium (*p *> 0.05).

### Statistical analysis

Fisher's exact test was applied to examine differences in allele frequencies (AF) between affected and unaffected individuals. A general linear model was used to test quantitative metabolic variables for differences between genotype groups assuming an additive (Add) model for *PKLR *rs3020781, and a dominant (Dom) model for *NOS1AP *rs7538490. Values of plasma glucose, serum insulin, HOMA-IR and BIGTT-AIR were logarithmically transformed before statistical analysis to obtain normal distribution. Adjustment for sex, age and BMI was applied when appropriate. All analyses were performed in RGui version 2.5.0 [28], and *p*-values < 0.05 were considered significant. Statistical power was determined using the CaTS power calculator version 0.0.2. A test for homogeneity between the population-based Inter99 cohort, the T2D patients and the population-based sample from Steno Diabetes Center and the ADDITION Denmark screening study cohort, was performed by means of the Mantel-Haenszel method (fixed effects model) for both genotypes, revealing no significant heterogeneity between study groups (rs3020781: *p *= 0.8, rs7538490: *p *= 0.4).

## Results and discussion

The minor allele frequencies (MAF) of the *PKLR *rs3020781 C-allele and the *NOS1AP *rs7538490 T-allele were 26.2% and 28.0%, respectively, and comparable to the 32.5% and 29.7%, reported for the HapMap CEU population. Using the population-based Inter99 cohort as reference the prevalence of T2D is estimated to 6% in the Danish population of middle-aged people. Combining the four study samples, gives us a statistical power of 100% observing an association with T2D with a relative risk above 1.3, and a MAF as reported for the two variants assuming either an additive or a dominant model. The potential associations between *PKLR *rs3020781 and *NOS1AP *rs7538490 and T2D were evaluated in case-control studies including 8,410 and 8,447 individuals, respectively. No difference in allele frequencies between T2D patients and glucose-tolerant subjects were found for either SNP (rs3020781: *p*_AF _= 0.49, OR [95% CI] = 1.02 [0.96–1.10]; rs7538490: *p*_AF _= 0.84, OR [95% CI] = 0.99 [0.93–1.06]), Table 1.

**Table 1:** Case-control studies of *PKLR *rs3020781 and *NOS1AP *rs7538490 in relation to type 2 diabetes, overweight and obesity

***PKLR *rs3020781**							
	***n *** **(men/women)**	**TT** **(%)**	**TC** **(%)**	**CC** **(%)**	**MAF** **(95% CI)**	***p*_AF_**	**OR** **(95% CI)**

**NGT**	4,736 (2,209/2,527)	2,602 (55)	1,812 (38)	322 (7)	25.9 (25.0–26.8)	0.49	1.02 (0.96–1.10)
		
**T2D**	3,674 (2,187/1,487)	1,998 (54)	1,412 (39)	264 (7)	26.4 (25.4–27.4)		

**BMI < 25 (kg/m^2^)**	5,036 (2,111/2,925)	2,732 (54)	1,952 (39)	352 (7)	26.4 (25.5–27.2)		

**25 ≤ BMI < 30 (kg/m^2^)**	6,985 (4,359/2,626)	3,821 (55)	2,700 (39)	464 (6)	26.0 (25.2–26.7)	0.49	0.98 (0.92–1.04)

**BMI ≤ 30 (kg/m^2^)**	4,780 (2,467/2,313)	2,612 (55)	1,845 (38)	323 (7)	26.2 (25.3–27.1)	0.81	0.99 (0.93–1.06)

***NOS1AP *rs7538490**							

	***n *(men/women)**	**CC** **(%)**	**CT** **(%)**	**TT** **(%)**	**MAF** **(95% CI)**	***p*_AF_**	**OR** **(95% CI)**

**NGT**	4,755 (2,218/2,537)	2,479 (52)	1,914 (40)	362 (8)	27.7 (26.8–28.7)	0.84	0.99 (0.93–1.06)
		
**T2D**	3,692 (2,200/1,492)	1,954 (53)	1,439 (39)	299 (8)	27.6 (26.6–28.6)		

**BMI < 25 (kg/m^2^)**	5,064 (2,128/2,936)	2,635 (52)	2,013 (40)	416 (8)	28.1 (27.2–29.0)		

**25 ≤ BMI < 30 (kg/m^2^)**	7,030 (4,384/2,646)	3,628 (51)	2,857 (41)	545 (8)	28.1 (27.3–28.8)	0.48	0.95 (0.83–1.09)

**BMI ≥ 30 (kg/m^2^)**	4,819 (2,490/2,329)	2,517 (52)	1,923 (40)	379 (8)	27.8 (26.9–28.7)	0.68	0.99 (0.93–1.05)

Data are number of individuals, divided into genotype groups (% in each group), and frequencies of the minor allele (MAF) in percentages. Fisher's exact test was used to compare allele frequencies (*p*_AF_). The odds ratios (OR) and 95% confidence interval (CI) are given for comparison of allele frequency. NGT: individuals with normal glucose tolerance, T2D: type 2 diabetic patients.

Case-control studies comparing allele frequencies between body mass index (BMI) defined normal weight (BMI < 25 kg/m^2^) individuals and overweight (25 kg/m^2 ^≤ BMI < 30 kg/m^2^) or obese (BMI ≥ 30 kg/m^2^) individuals, respectively, were performed in the combined study sample including T2D patients. No statistically significant association with overweight or obesity were demonstrated for *PKLR *rs3020781 (overweight: *p*_AF _= 0.49, OR [95% CI] = 0.98 [0.92–1.04]; obesity: *p*_AF _= 0.81, OR [95% CI] = 0.99 [0.93–1.06]) nor for *NOS1AP *rs7538490 (overweight: *p*_AF _= 0.48, OR [95% CI] = 0.95 [0.83–1.09]; obesity: *p*_AF _= 0.68, OR [95% CI] = 0.99 [0.93–1.05]), Table 1. As pharmacological treatment can influence on BMI, we additionally performed case-control studies of overweight and obesity considering T2D patients and treatment-naïve indviduals separately, however, neither variant showed association with overweight or obesity when stratifying according to glucose tolerance status (data not shown).

Furthermore, we investigated *PKLR *rs3020781 and *NOS1AP *rs7538490 for influence on diabetes-related quantitative metabolic phenotypes in 5,590 and 5,630 treatment-naïve Danish people from the population-based Inter99 cohort, respectively. No association with plasma glucose or serum insulin levels at fasting, 30 or 120 min during an OGTT or with OGTT-derived surrogate indices of insulin sensitivity or beta-cell function was demonstrated, Table 2. To evaluate the effect of the variants in individuals without impaired blood glucose regulation, analyses of quantitative metabolic phenotypes were conducted in the population-based Inter99 cohort including only glucose-tolerant individuals (*n*_*PKLR *_= 4,248, *n*_*NOS1AP *_= 4,269). However, no significant differences in genotype distribution of the two SNPs were demonstrated (data not shown).

**Table 2:** Quantitative metabolic characteristics of 5,590 and 5,630 treatment-naïve individuals from the population-based Inter99 cohort, stratified according to the *PKLR *rs3020781 genotype and the *NOS1AP *rs7538490 genotype, respectively

	***PKLR *rs3020781**				***NOS1AP *rs7538490**			
	
	**TT**	**TC**	**CC**	***p*_Add_**	**CC**	**CT**	**TT**	***p*_Dom_**
*n* (men/women)	3,065 (1,544/1,521)	2,169 (1,062/1,107)	356 (180/176)		2,954 (1,474/1,480)	2,238 (1,109/1,129)	438 (221/217)	

Age (years)	46 ± 8	46 ± 8	45 ± 8		46 ± 8	46 ± 8	46 ± 8	

BMI (kg/m^2^)	26.2 ± 4.6	26.2 ± 4.5	25.9 ± 4.3	0.58	26.2 ± 4.5	26.3 ± 4.6	26.1 ± 4.6	0.52

**Plasma glucose (mmol/l)**								

Fasting	5.5 ± 0.8	5.6 ± 0.9	5.5 ± 0.6	0.88	5.5 ± 0.7	5.5 ± 0.8	5.6 ± 1.1	0.46

30-min	8.7 ± 1.9	8.7 ± 1.9	8.6 ± 1.9	0.31	8.7 ± 1.9	8.7 ± 1.9	8.7 ± 1.8	0.90

120-min	6.2 ± 2.2	6.2 ± 2.1	6 ± 2.0	0.33	6.2 ± 2.1	6.2 ± 2.2	6.3 ± 2.1	0.22

**Serum insulin (pmol/l)**								

Fasting	34 (23–50)	35 (24–52)	31 (24–47)	0.21	34 (24–51)	34 (23–51)	34 (24–51)	0.14

30-min	243 (173–350)	248 (177–355)	246 (176–355)	0.29	244 (175–351)	247 (176–354)	248 (173–360)	0.67

120-min	154 (93–253)	161 (99–258)	150 (91–234)	0.45	155 (95–251)	157 (97–257)	158 (103–256)	0.59

**Derived indices**								

BIGTT-S_i_	9.2 (6.4–12.1)	9.1 (6.2–11.9)	9.8 (7.1–12.4)	0.54	9.2 (6.5–12.1)	9.2 (6.2–12.1)	9.6 (6.0–12.1)	0.37

BIGTT-AIR	1,622 (1,282–2,083)	1,625 (1,290–2,058)	1,634 (1,280–2,092)	0.84	1,618 (1,276–2,048)	1,643 (1,301–2,118)	1,632 (1,310–2,125)	0.05

HOMA-IR	8.2 (5.6–12.6)	8.5 (5.7–13.4)	7.7 (5.5–11.4)	0.24	8.3 (5.7–12.9)	8.4 (5.5–13.1)	8.0 (5.6–12.6)	0.14

Data are means ± standard deviation or median (interquartile range). Values of plasma glucose, serum insulin, HOMA-IR and BIGTT-AIR were logarithmically transformed before statistical analysis to obtain normal distribution. All analyses of *PKLR *rs3020781 were made using an additive model (Add), while analyses of *NOS1AP *rs7539480 were made using a dominant model. Calculated *p*-values were adjusted for age and sex for BMI measures, for sex, age and BMI for serum insulin, plasma glucose and HOMA-IR, and for age for the BIGTT-S_i _and BIGTT-AIR index. HOMA-IR was calculated as fasting plasma glucose (mmol/l) multiplied by fasting serum insulin (pmol/l) divided by 22.5. BIGTT-S_i _and BIGTT-AIR were calculated as described [26].

Despite successful identification of several T2D susceptible genes only a small percentage of T2D heritability is explained, thus, more T2D genes are to be found.

Originally two clusters of SNPs located within the T2D linkage peak were identified by The International Type 2 Diabetes 1q Consortium to associate with T2D among a total of 5,285 SNPs tagging the linkage peak. The first cluster of 9 SNPs were located in a linkage disequilibrium (LD) region including *PKLR *while the second cluster of 4 SNPs resided within *NOS1AP*. Replication of such potential associations, in statistically well-powered studies, is essential to substantiate the initial findings. Therefore, we aimed specifically at replicating the strongest associations in the two clusters of SNPs within *PKLR *and *NOS1AP*, which are rs3020781 and rs7538490, respectively. However, we did not show any association with T2D for either of the two variants, despite having the statistical power to detect the reported effect sizes. Neither did we find an association with pertinent metabolic phenotypes, which could indicate an impaired blood glucose regulation ultimately leading to T2D.

From our studies we can exclude rs3020781 and rs7538490 as T2D susceptibility variants in the Danish population, but *PKLR *and *NOS1AP *may still represent true T2D susceptibility loci. That *PKLR *represents a true T2D candidate gene, is supported by a study analysing two SNPs (rs1052176 and rs1052177) within *PKLR*, both showing association with T2D and both being in perfect LD with rs3020781 according to HapMap [21]. HapMap further outlined that rs3020781 is located at the border of a LD block near a recombination hotspot. Therefore, if the LD pattern is slightly shifted in our population, compared to the populations in which association is observed, rs3020781 may fail as a marker for the functional variant. Similar may be true for rs7538490, as LD is sparse in the region where *NOS1AP *is located. Thus, different LD patterns could explain the lack of association between rs3020781 and rs7538490 and T2D in our population.

In regards to the identification of T2D susceptibility genes, the linkage analysis, used for the identification of *PKLR *and *NOS1AP*, has been less successful due to inconsistent replication.

However, genome-wide association (GWA) studies have added to progress in finding common T2D susceptible gene variants with modest impact on diabetes risk [29-34], with the identification of non-obvious biological candidate genes and where replication have been predominantly successful [35-37]. We have investigated results of available data from GWA studies in web-based databases, but neither *PKLR *nor *NOS1AP *were among the high priority candidate genes as estimated from genome-wide significance levels [29,30]. Two markers in LD with *PKLR *rs3020781 were available in the public GWA data, however, none of these associated with T2D. No markers were available for *NOS1AP *rs7538490 [29]. The lack of association could either be due to small effect sizes, or the possibility that the variants represent false positive findings, thus explaining our failure to demonstrate an association.

## Conclusion

In statistically well-powered case-control studies and in studies of pertinent quantitative phenotypes we failed to validate the proposed association of the C-allele of rs3020781 and the T-allele of rs7538490 with T2D or intermediate phenotypes.

## Abbreviations

Add: additive model; AF: allele frequency; BIGTT-AIR: BIGTT acute insulin response; BIGTT-S_i_: BIGTT insulin sensitivity index; BMI: body mass index; CAPON: nitric oxide synthase 1 (neuronal) adaptor protein; CI: confidence interval; Dom: dominant model; GWA: genome-wide association; HOMA-IR: homeostasis model assessment of insulin resistance; LD: linkage disequilibrium; MAF: minor allele frequency; NGT: normal glucose tolerance; OGTT: oral glucose tolerance test; OR: odds ratio; SNP: single-nucleotide polymorphism; T2D: type 2 diabetes

## Competing interests

KBJ and OP hold stock in Novo Nordisk and have received lecture fees from pharmaceutical companies. All other authors declare that there is no competing interest associated with this manuscript.

## Authors' contributions

The original hypothesis was conceived by CHA and MSM and approved by OP and TH. Detail planning of analyses and study design was performed by CHA, MSM and approved by OP and TH. TJ, KBJ, TL, AS, OP and TH contributed to the epidemiological part of the recruitment of study populations. CHA, MSM, KA, LH, OP and TH contributed to the preparation of study populations for statistical analyses. Statistical analyses were performed by CHA and MSM. All authors contributed the interpretation of data. The first manuscript was written by CHA and MSM and the final draft was finalised by CHA, MSM, OP and TH. All authors revised the manuscript and contributed to the discussion of the results.

## Acknowledgements

The authors wish to thank A. Forman, I.-L. Wantzin and M. Stendal for technical assistance and G. Lademann for secretarial support. The study was supported by grants from the Danish Health Research Council, The European Union (EUGENE-2, grant no LSHM-CT-2004-512013), the FOOD Study Group/the Danish Ministry of Food, Agriculture and Fisheries and Ministry of Family and Consumer Affairs (grant no. 2101-05-0044), Novo Nordisk A/S Research & Development Corporate Research Affairs, the Danish Ministry of Science Technology and Innovation, the Faculty of Health Sciences of Aarhus University, the Danish Clinical Intervention Research Academy, and the Danish Diabetes Association. The ADDITION trial was supported by the National Health Services in the counties of Copenhagen, Aarhus, Ringkøbing, Ribe and South Jutland in Denmark, Danish Research Foundation for General Practice, Danish Centre for Evaluation and Health Technology Assessment, The diabetes fund of the National Board of Health, The Danish Medical Research Council, The Aarhus University Research Foundation and Novo Nordisk Foundation. Furthermore the ADDITION trial has been given unrestricted grants from Novo Nordisk A/S, Novo Nordisk Scandinavia AB, ASTRA Denmark, Pfizer Denmark, GlaxoSmithKline Pharma Denmark, SERVIER Denmark A/S and HemoCue Denmark A/S.

## Pre-publication history

The pre-publication history for this paper can be accessed here:



## Supplementary Material

Additional file 1 **Supplementary table 1.** Click here for file
