# Supplementary material for: Lack of association between PKLR rs3020781 and NOS1AP rs7538490 and type 2 diabetes, overweight, obesity and related metabolic phenotypes in a Danish large-scale study: case-control studies and analyses of quantitative traits
Source: BMC Med Genet. 2008 Dec 26;9:118. doi: 10.1186/1471-2350-9-118 (PMC2654670; doi:10.1186/1471-2350-9-118)
Supplement: Additional file 1 — Supplementary table 1. [file 1471-2350-9-118-S1.doc]

Supplementary Table 1

|  | **Inter99 population-based cohort** | **T2D patients from Steno Diabetes Center** | **Population-based study sample from Steno Diabetes Center** | **ADDITION Denmark screening study cohort** |
| --- | --- | --- | --- | --- |
| *n*_total_  (men/women) | 6,510  (3,168/3,342) | 2,125  (1,294/831) | 978  (472/506) | 8,644  (4,716/3,928) |
| Age (years) | 46 ± 8 | 62 ± 11 | 59 ± 8 | 60 ± 7 |
| BMI (kg/m^2^) | 26.3 ± 4.6 | 30.0 ± 5.6 | 26.5 ± 4.2 | 28.6 ± 4.9 |
| ***PKLR* rs3020781** | | | | |
| **Genotyped** | **5,962** | **1,873** | **599** | **8,367** |
| BMI Controls | 2,617 | 321 | 260 | 1,838 |
| BMI Overweight | 2,323 | 703 | 248 | 3,711 |
| BMI Obese | 1,022 | 849 | 91 | 2,818 |
| NGT | 4,248 | - | 488 | - |
| T2D  (treated/non-treated) | 328  (107/221) | 1,752  (1,752/0) | 18  (7/11) | 1,576  (0/1,576) |
| ***NOS1AP* rs7538490** | | | | |
| **Genotyped** | **6,008** | **1,874** | **596** | **8,435** |
| BMI Controls | 2,630 | 322 | 254 | 1,858 |
| BMI Overweight | 2,336 | 705 | 253 | 3,736 |
| BMI Obese | 1,042 | 847 | 89 | 2,841 |
| NGT | 4,269 | - | 486 | - |
| T2D  (treated/non-treated) | 337  (108/229) | 1753  (1,753/0) | 18  (7/11) | 1,584  (0/1,584) |

The combined study sample comprising four different study groups, showing data for mean age and BMI ± standard deviation, and the distribution of individuals divided according to BMI (controls BMI < 25 kg/m^2^, overweight 25 ≤ BMI < 30 kg/m^2^ and obese BMI ≥ 30 kg/m^2^) and glucose tolerance status (NGT: individuals with normal glucose tolerance, T2D: type 2 diabetics) for the two variants.
